# Whole chloroplast genome sequence of a subtropical tree *Eriobotrya bengalensis* (Rosaceae)

**DOI:** 10.1080/23802359.2020.1714510

**Published:** 2020-01-16

**Authors:** Jian Xu, Cheng Liu, Yaxuan Xin, Jing Xin, Li Feng, Fayu Feng, Linyi Yang, Zhenghai Sun

**Affiliations:** a South and Southeast Asia Joint R&D Center of Economic Forest Full Industry Chain, Southwest Forestry University, Kunming, China;; b International Technologial Cooperation Base of High Effective Economic Forestry Cultirating, Southwest Forestry University, Kunming, China;; c Key Laboratory of Forest Resources Conservation and Utilization in the Southwest Mountains of China Ministry of Education, Southwest Forestry University, Kunming, China

**Keywords:** *Eriobotrya*, chloroplast genome, phylogenetic analyses

## Abstract

*Eriobotrya bengalensis* (Roxb.) is a subtropical plant under the family Rosaceae with high economic and medicinal value. The whole chloroplast genome of *E. bengalensis* was sequenced to better understand its phylogenetic position relative to other Rosaceae species. The total length of the *E. bengalensis* chloroplast genome was 159,270 bp, which was composed of a large single-copy (LSC) region of 87,362 bp, a small single-copy (SSC) region of 19,184 bp, and a pair of inverted repeats (IRs) with a length of 26,362 bp separated by LSC and SSC. The total G + C content of the whole chloroplast genome was 36.7%. Phylogenetic analysis of maximum likelihood (TVM + F+R2) was completed using 15 complete chloroplast genomes of Rosaceae species. The results of phylogenetic analysis show that sisterhood exists in *E. bengalensis* with nine other species of *Eriobotrya*.

*Eriobotrya bengalensis* (Roxb.) Hook. f. is a valuable genetic resource for breeding spring-flowering cultivars, which bloom in March and April (Wang et al. 2017). Relevant research shows that the distribution range of different species of *Eriobotrya* varies. The widely distributed species include *E. fragrans* and *E. deflexa*, whereas the distribution of *E. bengalensis* and *E. malipoensis* is limited (Lin et al. 2004). The whole chloroplast genome of *E. bengalensis* was used to reconstruct a phylogenetic tree based on high-throughput sequencing approaches to better understand the relationship of *E. bengalensis* with other *Eriobotrya* species.

Fresh and young leaves (5 g) of *E. bengalensis* were obtained from Xishuangbanna Tropical Botanical Garden (101.16°E, 21.55°N; 566 m above sea level) for DNA extraction by the modified CTAB method (Shen et al. 2016). The voucher specimen was deposited at the Key Laboratory of Forest Resources Conservation and Utilization in the Southwest Mountains of China Ministry of Education, Southwest Forestry University (Accession Number. SWFU-SY35370). The complete chloroplast genome was sequenced following Yang et al. (2014), and the next-generation was sequenced with nine pairs of universal primers via long-range PCR amplification. The whole chloroplast genome was assembled with GetOrganelle (Jin et al. 2018), whereas the whole chloroplast genome was used for initial chloroplast genome annotation in Geneious R8.1.9 (Biomatters Ltd, Auckland, New Zealand), using the publicly available chloroplast genome of *E. fragrans* (Accession Number: LAU10001) (Dong et al. 2019) and *E. malipoensis* (Accession Number: LAU10002) (Qu et al. 2019) as a reference.

The chloroplast genome of *E. bengalensis* (LAU10004) with a length of 159,270 bp was 16 and 361 bp smaller than those of *E. fragrans* (159,286 bp, LAU10001) and *E. henryi* (159,631 bp, MN577880), respectively. The chloroplast genome of *E. bengalensis* was also 60 and 2,276 bp larger than those of *E. cavaleriei* (159,210 bp, MK920283) and *E. malipoensis* (156,994 bp, MN577881), respectively. The complete chloroplast genome of *E. bengalensis* was composed of a large single-copy (LSC) region of 87,362 bp, a pair of inverted repeats (IRs) of 26,362 bp, and a small single-copy (SSC) region of 19,184 bp. The overall G + C content was 36.7% (LSC, 34.4%; IRs, 30.5%; SSC, 42.6%). The *E. bengalensis* chloroplast genome encodes 134 genes, including 89 protein-coding genes, 37 tRNA genes, and 8 rRNA genes.

We reconstructed a phylogenetic tree (Figure 1) based on 14 published chloroplast genome sequences of the family Rosaceae, to acknowledge the evolutionary relationship between *E. bengalensis* and other *Eriobotrya* species with the published chloroplast. In addition, *Pyrus ussuriensis* (Accession Number: MK172841) was treated as an out-group, which was aligned by MAFFT version 7 (Katoh and Standley 2013). IQ-TREE 1.6.7 with 1000 bootstrap replicates was employed (Nguyen et al. 2015), and maximum likelihood (ML) phylogenetic analyses were carried out based on the TVM + F + R2 model. The ML phylogenetic tree showed 48–100% bootstrap values at each node, which confirmed that sisterhood exists between *E. bengalensis* and nine other *Eriobotrya* species with the published chloroplast.

**Figure 1.**
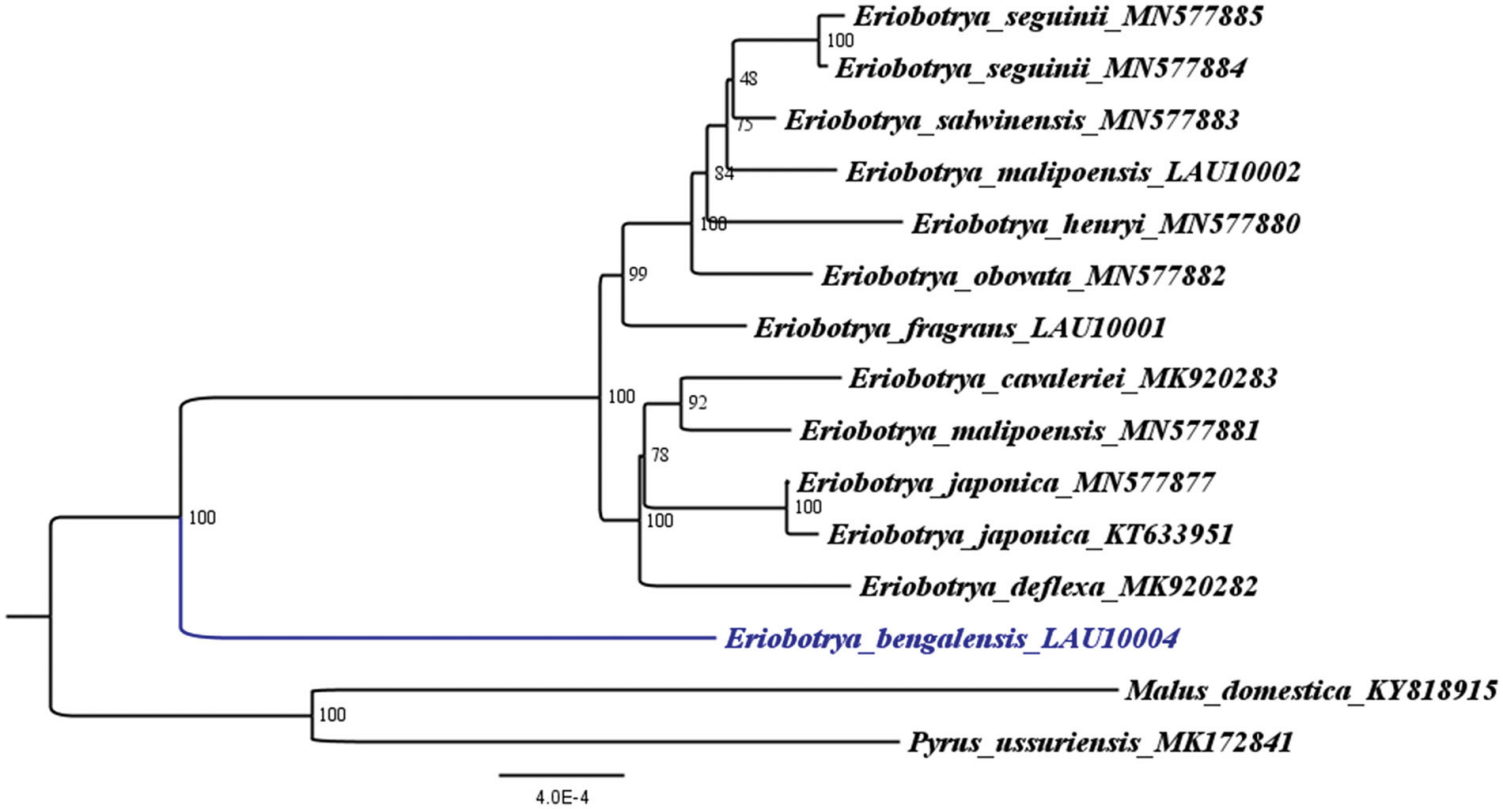
The ML phylogenetic tree for *E. bengalensis* based on other 14 species (12 in *Eriobotrya*, 1 in *Malus*, 1 in *Pyrus*) chloroplast genomes.

## Data Availability

The chloroplast data of the *E. bengalensis* will be submitted to Rosaceae Chloroplast Genome Database (https://lcgdb.wordpress.com). Accession numbers are LAU10004
